# Mechanical Analysis of Needles Used in Ultrasound-Guided Musculoskeletal Interventions

**DOI:** 10.33549/physiolres.935721

**Published:** 2026-02-01

**Authors:** Tomas NOVOTNY, Daniel HADRABA, Kamal MEZIAN, Levent ÖZÇAKAR

**Affiliations:** 1 Department of Orthopaedics, Faculty of Health Studies, Jan Evangelista Purkyne University in Usti nad Labem and Masaryk Hospital, Usti nad Labem, Czech Republic; 2 Department of Orthopaedic Surgery, Faculty of Medicine in Hradec Kralove, Charles University, Hradec Kralove, Czech Republic; 3 Laboratory of Advanced Microscopy and Data Analyses, Institute of Physiology of the Czech Academy of Sciences, Prague, Czech Republic; 4 Department of Rehabilitation Medicine, First Faculty of Medicine, Charles University and General University Hospital, Prague, Czech Republic; 5 Department of Physical and Rehabilitation Medicine, Hacettepe University Medical School, Ankara, Turkey

**Keywords:** Interventional ultrasound, Needle, Buckling strength

## Abstract

We performed a mechanical analysis of the commonly used needles for ultrasound-guided interventions in the musculoskeletal system. Specifically, focusing on the ability to absorb potential physical loads and the degree of deformation during the procedure, the needle gauge best suited for those procedures is evaluated. A customized tensile-compression device was used for an experimental buckling strength test on three commonly used needle types with specific gauge sizes. The loss of structural integrity, loss of needle stability, and buckling load were modeled also theoretically using finite element analysis software. Theoretical software needle buckling analysis detected the load for the first buckling mode of the needle, when the highest value was reached for the G20 needle with the load 18.8 N. The load for G21 needle was 9.7 N and for G23 8 N. Experimental data with customized tensile-compression device aligned theoretical data when the highest value was reached for the G20 needle with the load 19.7±1.9 N. The load for G21 needle was 10.6±2.7 N and for G23 7.9±0.7 N. Theoretical and practical experiments have shown that the standard G20 needle model exhibits the highest mechanical tolerance for potential interventions in the musculoskeletal system.

Owing to several advancements, the use of ultrasound (US) guidance for musculoskeletal interventions has largely/recently evolved [1–4]. Studies indicated that US-guided interventions are generally more accurate and effective than their non-guided versions [5]. Herewith, various efforts have been proposed to enhance the efficiency of those interventions; both as regards the technical issues (US, probe, etc.) and the clinical skills (knowledge, training, etc.) [6].

A possible pitfall when using US guided interventions is poor needle visibility due to deflection error as a result of needle deformation. The length of the needle is usually chosen according to the depth of the targeted structure, with the medium-length needles (50–70 mm) being commonly used [7]. Of note, real-time US guidance (using both in-plane and out-of-plane techniques) typically requires the intervention to occur at a greater distance from the puncture site. The longer the needles are, due to their biomechanical properties, the more they become prone to deformation during the intervention. Further, during musculoskeletal (MSK) interventions, direct contact with bones or stiff soft tissues (e.g. calcification), could also lead to secondary needle deformation.

Previous literature has primarily focused on improving echogenicity and, to a lesser extent, on modeling needle deformation [8–10]. However, no study to date has conducted a systematic buckling load analysis of standard needles routinely used in MSK interventions. Accordingly, the purpose of this study was to assess, through a biomechanical experiment, the ability of various commonly used needle gauges to endure longitudinal mechanical pressure and resistance to the formation of residual needle deformation. Eventually, possible identification of the most appropriate needle(s) for MSK interventions was aimed.

The needle line was B Braun™ Sterican™ Single-use. We tested three sizes of routinely used needles with specific gauge size (G), length (l), external diameter (ØD) and internal diameter (Ød). First needle was gauge size G20 (yellow) (l=70 mm, ØD=0.910 mm, Ød=0.650 mm), second was G21 (green) (l=80 mm, ØD=0.820 mm, Ød=0.565 mm), and third was G23 (blue) (l=60 mm, ØD=0.640 mm, Ød=0.385 mm).

The needle theoretical buckling strength, the stress level at which the needle suddenly deforms under compression, was established using a conservative approach of linear eigen buckling analysis in finite element analysis (FEA) software ANSYS Mechanical. The needle geometry was designed according to the datasheet. The needle material is standard 316 Stainless Steel Tubing. The degrees of freedom for the needle shell were constrained at fixed deformation U_x_, U_y_, U_z_ and free rotation R_x_, R_y_, R_z_ at the bevel end. The other side of the needle had also fixed deformation in U_x_, U_y_ but free in U_z_ direction. The rotations are again free. The load is applied in the z direction for the analysis (Fig. 1a). The value for the first buckling mode is stated in this paper. The experimental buckling strength was measured using a customized tensile-compression device (Fig. 1b). The compression test was performed at 0.5 mm/s where the force and position of the jaws was recorded. The buckling load was established at a point where the slope in the force-deflection curve changes abruptly.

Theoretical software needle buckling analysis detected the load for the first buckling mode of the needle, when the highest value was reached for the G20 needle with the load 18.8 N. The load for G21 needle was 9.7 N and for G23 8 N. Experimental data with customized tensile-compression device aligned theoretical data when the highest value was reached for the G20 needle with the load 19.7±1.9 N. The load for G21 needle was 10.6±2.7 N and for G23 7.9±0.7 N. The loss of structural integrity calculated in ANSYS in details is given in Table 1. The simulation in ANSYS is demonstrated in Figure 1c. The details on loss of needle stability as measured experimentally is given in Table 1. The results are aligned with the FE analysis in ANSYS.

This study examined the deformability of needles routinely used for ultrasound-guided musculoskeletal interventions when exposed to mechanical loading. Among the tested gauges, the G20 needle showed the highest resistance to buckling.

Our results are consistent with the findings of Swenson *et al.* [11] who compared a fenestrated G20 needle with a conventional G22 design and reported significantly greater buckling strength and stiffness in the larger-diameter needle. Interestingly, even our G23 needle demonstrated a higher buckling load (7.9±0.7 N) than the G22 needle in their series, most likely reflecting differences in design, manufacturer, and testing protocol. Despite these differences, both studies confirm the same principle that larger-diameter needles (i.e. lower gauge number) are less prone to buckling and therefore provide greater mechanical stability.

Similar conclusions can be drawn from biomechanical modeling studies. Rossa and colleagues [8], as well as Moreira and Misra [9], demonstrated that flexible needles are more likely to deviate from the planned trajectory, leading to measurable targeting errors. Their work focused on gradual deflection within soft tissues rather than sudden buckling, yet the underlying message is similar i.e. needle stiffness is a key determinant of procedural accuracy. In this context, it is useful to distinguish between the two phenomena; buckling refers to the abrupt loss of stability under axial compression, whereas deflection describes the progressive deviation of the needle tip due to tissue resistance. Our study specifically addressed the buckling strength, providing an experimental and theoretical evaluation that complements previous deflection models.

Another aspect of needle performance relates to visibility. Markham *et al.* [12] showed that surface texturing could increase US signals return up to sevenfold compared with a smooth surface, while Abbal and Hebard [13,14] demonstrated that echogenic coatings improve tip visibility at steep insertion angles. These studies highlight that echogenicity and mechanical stability are not separate issues but rather complementary properties. Both are essential to ensure safe and precise needle placement under US guidance.

From a clinical perspective, needle gauge selection has direct consequences for safety and accuracy. Greater mechanical stability reduces the likelihood of unwanted bending or misdirection, particularly when the needle encounters resistance from bone, calcified deposits, or fibrotic tissue. At the same time, larger needles are easier to visualize in US images, further supporting precise guidance. However, thinner needles may still be preferable in certain settings, for example when patient comfort, tissue fragility, or the need for maneuverability in tight anatomical spaces takes precedence. For this reason, needle choice should never be reduced to a single parameter.

This study has limitations. Only three needle types from a single manufacturer were tested, which restricts the generalizability of the results. All experiments were conducted under *in-vitro* conditions and do not fully replicate the complexity of living tissues, including variable resistance, dynamic operator forces, or repeated manipulation. Moreover, we focused exclusively on buckling strength and did not evaluate other mechanical features such as torsional resistance, fatigue under repeated loading, or the combined impact of echogenic modifications. Future work should therefore include a wider spectrum of needles, test conditions closer to the clinical environment, and integrated evaluation of both mechanical and imaging properties.

The results indicated that the gauge size G20 needle exhibited the greatest stability under mechanical loads. As a general framework, larger diameter needles combine greater stability with improved US visibility, supporting both safety and accuracy. At the same time, needle properties must always be chosen case by case, considering the anatomical location, the intended technique, and the characteristics of the target tissue.

## Figures and Tables

**Fig. 1:**
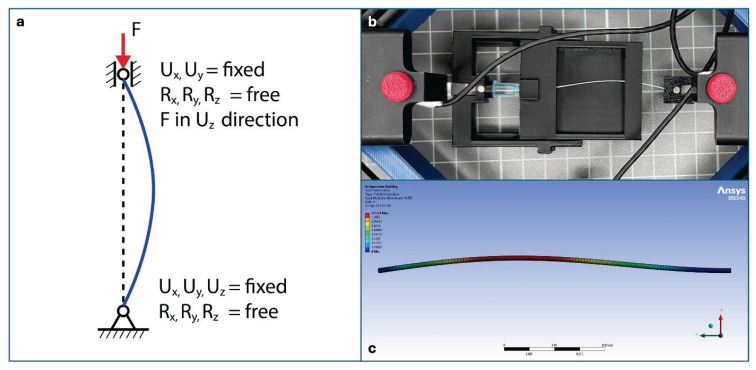
(**a**) The end conditions applied for the needle finite element analysis in ANSYS Mechanical software; (**b**) The snap of the needle test that theoretically represents the end conditions in Figure 1a; (**c**) Resulting deformation of the eigen buckling in ANSYS Mechanical software displaying the first mode (B Braun™ Sterican™ Single-use, gauge size G20, length 80 mm).

**Table 1:** The buckling load for the measured needles and on the experimental data.

*Needle Type*	Load for First Buckling Mode (Measured Needles)
*G20*	18.8 N
*G21*	9.7 N
*G23*	8.0 N

*Needle Type*	**Load for First Buckling Mode (Theoretical Experiment)**

*G20*	19.7 ± 1.9 N
*G21*	10.6 ± 2.7 N
*G23*	7.9 ± 0.7 N
